# Interval training based on ventilatory anaerobic threshold increases cardiac vagal modulation and decreases high-sensitivity c-reative protein: randomized clinical trial in coronary artery disease

**DOI:** 10.1590/bjpt-rbf.2014.0124

**Published:** 2015-10-09

**Authors:** Nayara Y. Tamburus, Roberta F. L. Paula, Vandeni C. Kunz, Marcelo C. César, Marlene A. Moreno, Ester da Silva

**Affiliations:** 1 Núcleo de Pesquisa em Exercício Físico, Departamento de Fisioterapia, Universidade Federal de São Carlos (UFSCar), São Carlos, SP, Brazil; 2 Faculdade de Ciências da Saúde, Universidade Metodista de Piracicaba (UNIMEP), Piracicaba, SP, Brazil; 3 Centro Universitário Adventista (UNASP), Engenheiro Coelho, SP, Brazil; 4 Laboratório de Performance Humana, Faculdade de Ciências da Saúde, UNIMEP, Piracicaba, SP, Brazil

**Keywords:** physical therapy, coronary disease, exercise, C-reactive protein, heart rate variability

## Abstract

**Background:**

Autonomic dysfunction and inflammatory activity are involved in the development and progression of coronary artery disease (CAD), and exercise training has been shown to confer a cardiovascular benefit.

**Objective:**

To evaluate the effects that interval training (IT) based on ventilatory anaerobic threshold (VAT) has on heart rate variability (HRV) and high-sensitivity C-reactive protein (hs-CRP) levels, as well as the relationship between both levels, in patients with CAD and/or cardiovascular risk factors (RF).

**Method:**

Forty-two men (aged 57.88±6.20 years) were divided into two training groups, CAD-T (n= 12) and RF-T (n= 10), and two control groups, CAD-C (n= 10) and RF-C (n=10). Heart rate and RR intervals in the supine position, cardiopulmonary exercise tests, and hs-CRP levels were measured before and after IT. HRV was analyzed by spectral and symbolic analysis. The CAD-T and RF-T underwent a 16-week IT program of three weekly sessions at training intensities based on the VAT.

**Results:**

In the RF-T, cardiac sympathetic modulation index and hs-CRP decreased (p<0.02), while cardiac parasympathetic modulation index increased (p<0.02). In the CAD-T, cardiac parasympathetic modulation index increased, while hs-CRP, systolic, and diastolic blood pressures decreased (p<0.02). Both control groups showed increase in hs-CRP parameters (p<0.02). There was a strong and significant association between parasympathetic and sympathetic modulations with hs-CRP.

**Conclusion:**

The IT program based on the VAT promoted a decrease in hs-CRP associated with improvement in cardiac autonomic modulation.

## Introduction

Autonomic dysfunction and increased circulating levels of inflammatory biomarkers are interrelated risk factors implicated in the etiology of coronary artery disease (CAD)^1^ and other cardiovascular diseases^2^. Recent studies have explored the link between the autonomic nervous system (ANS) and markers of inflammation^1^^,^ 3. These previous studies have proposed a relationship between increased high-sensitivity C-reactive protein (hs-CRP) levels and decreased vagal modulation in patients with CAD, which is also associated with an increased risk of cardiovascular events and progression of CAD^1^^,^ 3. On the other hand, it has been reported that the parasympathetic nervous system may inhibit inflammation by discharging acetylcholine and suppressing the synthesis and release of pro-inflammatory cytokines^4^.

Aerobic exercise training promotes positive autonomic adaptations^5^, which may be evidenced by an increase in cardiac parasympathetic modulation. Among several types of exercise protocol, interval training (IT) has gained considerable attention as a suitable exercise program for patients with CAD^6^^,^ 7. Several studies suggest that high-intensity IT promotes more evident cardiovascular adaptions than low and moderate intensity of exercise in patients with CAD^8^^,^ 9. Regarding autonomic adaptation, Currie et al.^7^ reported that neither moderate-intensity endurance exercise nor novel low-volume high-intensity interval exercise protocol programs promoted improvements in cardiac autonomic function in patients with CAD. In contrast, Munk et al.^10^showed that high-intensity IT leads to consistent favorable effects on heart rate variability (HRV).

Despite these findings, there is a scarcity of clinical data on the effects of IT at intensities close to the ventilatory anaerobic threshold (VAT) on autonomic modulation of heart rate (HR). Moreover, it is still unclear whether the increase in parasympathetic modulation is related to a reduction in inflammation after the IT program.

Therefore, the purpose of this study was to evaluate the effects that IT based on the VAT might have on HRV and hs-CRP levels, as well as the relationship between both parameters, in patients with CAD and/or cardiovascular risk factors (RF). We hypothesized that the proposed IT program would improve HRV indices, as evidenced by increased parasympathetic modulation and decreased sympathetic modulation, in association with a decrease circulating levels of hs-CRP.

## Method

### Subjects

The study was designed as a randomized clinical trial (ClinicalTrials.gov Identifier: NCT02313831). A sample of 139 male subjects was interviewed and considered eligible for the study based on the results of coronary angiography performed at Centro de Hemodinâmica do Hospital Santa Isabel, Piracicaba, SP, Brazil. All subjects were screened and classified as either CAD or RF according to the following criteria: CAD - subjects with angiographic evidence of ≥50% of stenosis in one or more major coronary vessels or previous coronary artery intervention, such as percutaneous coronary intervention (PCI) and coronary artery bypass graft (CABG); RF - subjects without stenosis in coronary vessels (angiographically documented) and without a history of previous myocardial infarction (MI), PCI or CABG. All subjects presented three or more cardiovascular RF, such as obesity (body mass index >30 kg/m^2)^, hypertension, diabetes mellitus (type 2 - nonusers of insulin), dyslipidemia, smoking, and sedentary lifestyle according to the International Physical Activity Questionnaire (IPAQ) version 6. The exclusion criteria consisted of MI<6 months, PCI and CABG<3 months, frequent arrhythmias, pulmonary disease, unstable angina, osteomuscular disorders, insulin-dependent diabetes mellitus, neoplasia, renal failure, and sequelae associated with stroke.

**Figure 1:**
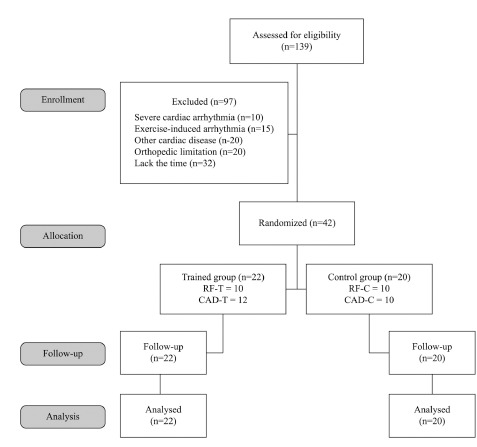
Flowchart showing patient participation in the study. CAD-T= trained group with patients with coronary artery disease; CAD-C= control group with patients with coronary artery disease; RF-T= trained group with patients with cardiovascular risk factors; RF-C= control group with patients with cardiovascular risk factors.

In this study, 42 men were selected (22 with CAD and 20 with only cardiovascular RF) according to the inclusion criteria. The flowchart of the progress of the study is presented in Figure 1, and subject characteristics are described in Table 1. All subjects performed a resting 12-lead electrocardiogram and a maximal or symptom-limited exercise test using the Bruce treadmill protocol under the supervision of a cardiologist with the objective of pre-exercise screening and risk stratification for physical activity. Following the exercise testing, a random number table was used to allocate the CAD subjects to trained group (CAD-T, n=12) or control group (CAD-C, n=10) and the RF subjects to trained group (RF-T, n=10) or control group (RF-C, n=10). 

**Table 1.** Baseline clinical characteristics of the trained and control groups.

**Variables**	**Trained**	**Control**		
	**RF (n=10)**	**CAD (n=12)**	**RF (n=10)**	**CAD (n=10)**
Age (years)	58.9±4.0	56.2±7.4	56.3±6.2	60.4±6.1
**Clinical characteristics - number of patients (%)**				
Myocardial infarction	0(0.0%)	4(33.3%)	0(0.0%)	2(20.0%)
Coronary artery bypass surgery	0(0.0%)	4(33.3%)	0(0.0%)	4(40.0%)
Percutaneous coronary intervention	0(0.0%)	8(66.6%)	0(0.0%)	6(60.0%)
**Medication - number of patients (%)**				
Beta blockers	4(40.0%)	7(58.3%)	4(40.0%)	5(50.0%)
ACE inhibitors	3(30.0%)	7(58.3%)	7(70.0%)	4(40.0%)
Hypolipidemic agents	5(50.0%)	10(83.3%)	2(20.0%)	8(80.0%)
Diuretics	1(20.0%)	3(25.0%)	2(20.0%)	2(20.0%)
Antiplatelet agents	4(40.0%)	10(83.3%)	6(60.0%)	9(90.0%)
Hypoglycemic agents	0(0.0%)	1(8.3%)	2(20.0%)	2(20.0%)
**Risk factor - number of patients (%)**				
Smoking	1(10.0%)	3(25.0%)	3(30.0%)	1(10.0%)
Sedentary lifestyle (IPAQ)	10(100.0%)	12(100.0%)	10(100.0%)	10(100.0%)
Dyslipidemia	7(70.0%)	10(83.3%)	3(30.0%)	9(90.0%)
Hypertension (140/90 mHg)	5(50.0%)	8(66.6%)	7(70.0%)	7(70.0%)
Obesity (BMI ≥ 30 kg/m^2)^	1(10.0%)	5(41.6%)	3(30.0%)	3(30.0%)
Diabetes mellitus	0(0.0%)	1(8.3%)	2(20.0%)	2(20.0%)
**Metabolic variables**				
Triglycerides (mg/dL)	119.9±59.1	154.2±70.0	138.8±44.4	153.1±74.0
Total cholesterol (mg/dL)	177.7±46.7	172.2±50.1	164.8±24.0	171.6±22.1
Fasting glucose (mg/dL)	93.2±7.5	98.2±10.9	90.0±10.0	97.2±12.1
HDL cholesterol (mg/dL)	48.1±20.1	40.7±10.5	39.9±11.6	39.1±9.8
LDL cholesterol (mg/dL)	97.2±29.5	105.6±34.3	100.7±17.9	98.7±26.4
**Ventilatory anaerobic threshold**				
VO_2_(mL.kg^-1^.min^-1)^	14.5±2.4	13.50±2.14	14.38±2.06	13.15±3.33
VO_2_ (L.min^-1)^	1.15±0.24	1.12±0.25	1.14±0.28	1.08±0.20
HR (bpm)	105.6±15.1	106.9±12.7	115.7±22.3	103.4±24.4
Workload (W)	82.6±28.0	80.2± 6.8	84.3±24.3	81.7±17.3


Values are represented as mean ± SD, except where indicated. CAD = coronary artery disease; RF = risk factor; ACE = angiotensin converting enzyme; IPAQ = International Physical Activity Questionnaire; HDL = high-density lipoprotein; LDL = low-density lipoprotein; VO2 = oxygen consumption; HR = heart rate; bpm = beats per minute; W = watts.

All subjects signed an informed consent form prior to participating in the study, which was approved by the Ethics Committee of Universidade Metodista de Piracicaba (UNIMEP), Piracicaba, SP, Brazil (Protocol 34/12).

### Experimental procedures

All experimental procedures were performed in the morning. The room temperature and relative air humidity of the testing laboratory were kept at 23°C and between 40% and 60%, respectively. Before the experimental procedures, all subjects were familiarized with the equipment and experimental protocol to reduce anxiety. They were then instructed to abstain from stimulants (coffee, tea, soft drinks) and alcoholic beverages, avoid exhausting physical activity in the 24 h prior to the test, and have a light meal at least 2 h before the experiment.

### Blood plasma parameters

After an overnight 12-h fast, venous blood samples were drawn to analyze the following parameters: fasting glucose, total cholesterol (using the autoanalyzer method), high-density lipoprotein (HDL) and low density lipoprotein (LDL) cholesterol (using enzymatic colorimetry), triglycerides (using automated enzymatic methods), and hs-CRP (with the nephelometric method).

### Measurement and analysis of HRV

Each subject's HR and RR intervals were measured for 15 minutes with a digital telemetry system consisting of a transmitter placed on the chest and a HR monitor (Polar^Ò^ S810i; Polar Electro Oy, Kempele, Finland) previously validated by Gamelin et al.^11^. The measurements were obtained with the subjects in the supine position while breathing spontaneously (i.e. ≈ 14 breaths per minute).

After transferring the data to the computer, 300 consecutive beats were selected after the length of greatest stability was chosen from the central region of the time series. The initial and final RR intervals were discarded. The HRV was evaluated based on spectral^12^ and symbolic analyses^13^. The same sequence was used for both the spectral and the symbolic analysis. Detailed procedures for HRV analysis were as previously described^12^^,^ 13.

### Cardiopulmonary exercise test (CPET)

Each subject underwent submaximal CPET on a cycle ergometer with an electromagnetic brake (Corival V2, Lode B.V., Groningen, The Netherlands) to determine the VAT before IT. Workload increments were determined for each subject according to the formula proposed by Wasserman et al.^14^ (Workload increase (W) = [(height - age) x 20] - [150 + (6 x body mass)]/100). CPET was performed with 1 min baseline; 4 min unloaded cycling, followed by the workload increments. The test was interrupted when the patients had symptom- and/or sign-limited HR (pallor, unusual sweating patterns, nausea, vomiting, physical exultation, dimmed or blurred vision, dyspnea, and abnormal response of SBP and HR in progressive workloads) or attained submaximal HR (85% of maximum HR)^15^. Ventilatory and metabolic measurements were obtained on a breath-by-breath basis using a metabolic analyzer (CPX-D, Medical Graphics, St. Paul, MN, USA). HR was monitored throughout the test using a 12-lead electrocardiogram (Welch Allyn CardioPerfect Workstation, Skaneateles Falls, NY, USA).

The VAT was determined from a loss of parallelism between VO_2_ (oxygen consumption) and VCO_2_ (carbon dioxide output) by three properly trained observers, as previously described by Zamunér et al.^16^ and Higa et al.^17^.

### Interval training program

**Figure 2:**
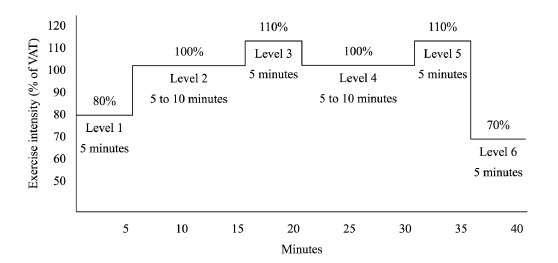
Visual presentation of intensity levels and duration performed during of interval training. VAT= ventilatory anaerobic threshold.

The IT program was individualized and administered three times per week (on alternate days) for a 16-week period. Each exercise session lasted approximately 60 min and was divided into three parts as follows: **1) Warm-up** (10 min) including stretching, calisthenics, and low-intensity exercises (walking); **2) Exercise training protocol** (Figure 2) performed on a stationary cycle ergometer (30-40 min)^18^^,^ 19 and was divided into 6 steps: **Step 1** - 5-min at moderate intensity with the aim of reaching 80% of the workload attained at VAT; **Step 2 and 4** - 5-min and progressing up to 10-min at moderate-high intensity with the aim of reaching 100% of the workload attained at VAT; **Step 3 and 5** - 5-min at moderate to high intensity with the aim of reaching 110% of the workload attained at VAT; **Step 6 -** 5-min at moderate intensity with the aim of reaching 70% of workload attained at VAT. The moderate and moderate-high intensities were comprised, thus eliciting the aerobic and the anaerobic metabolism. **3) Cool-down** (10 min) consisted of stretching and respiratory exercises to allow the BP and HR values to return to their near-basal values. Polar HR monitors (Polar^Ò^ S810i; Polar Electro Oy, Kempele, Finland) were worn during each exercise session to ensure that patients exercised at the targeted intensity. 

The intensity of the physical training was adjusted on a monthly basis, following the methodology proposed by Sirol et al.^18^ and Pithon et al.^19^, by determining the anaerobic threshold from the HR response. HR response was analyzed during discontinuous exercise testing on cycle ergometer in each workload applied. HR data behavior was analyzed based in mathematical and statistical models, whereby evaluated the loss of stability of the HR response (decreasing, stable, or increasing response as a function of time). A positive slope and statistically significant was considered as workload at VAT level, which has been attributed to the beginning of the predominance of sympathetic activation. Thus, the physical training intensity levels were adjusted based on the HR data at the power output level corresponding to the positive slope of HR.

### Statistical analysis

Based on the pilot study (n=6), the sample size was calculated using GPower software (version 3.1) considering a 5% chance of type I error, a 2-sided test, and a power of 80% power, according to Lara Fernandes et al.^20^. The outcome variable considered for the sample size calculation was hs-CRP, based on analysis of variance (repeated measures; within between interaction), which provided an effect size (ES) of 0.77. A final sample of 10 patients in each group was suggested. According to the frequency distribution analysis (Shapiro-Wilk test), the data did not show a normal distribution. Baseline clinical characteristics are presented as means ± standard deviation. Hemodynamic variables at rest, HRV indices, and hs-CRP levels are presented as medians (interquartile range). The Mann-Whitney test was performed for intergroup comparison, and the Wilcoxon test was used for intragroup comparison. To adjust for multiple comparisons, the Bonferroni correction was used at a signiﬁcance level of p<0.02. The ES for each mean difference was calculated using Cohen's d^21^. The thresholds used for interpretation were as follows: values >0.2 to 0.5 = small ES, >0.5 to 0.8 = medium ES, and >0.8 = large ES.

Analysis of covariance (ANCOVA) and linear regression were used to assess the relationship between HRV indices and hs-CRP (dependent variables), as well as covariates (independent variables) such as clinical variables, use of medication, and risk factors (Table 1). The correlation between HRV indices and hs-CRP was analyzed using partial correlation adjusted for the following covariates: clinical variables, use of medication, and risk factors, based on their significance in the covariance test (ANCOVA). The correlation coefficient was calculated (r=0.40 to 0.50, weak correlation; r=0.6 to 0.7, moderate correlation; r=>0.8, strong correlation). Significance was established at the level of p<0.05 (α=5%). 

All statistical analyses were performed using the software packages STATISTICA for Windows 7.0 and R version 2.9.0 for Windows.

## Results

There were no changes in anthropometrics characteristics at baseline and after follow-up in trained and control groups. At baseline, the resting systolic BP, diastolic BP and HR did not differ significantly among trained and control groups (p>0.02). After IT, a significant decrease in resting systolic and diastolic BP values was noted in the CAD-T group (p<0.02). Moreover, systolic (p<0.02) and diastolic BP (p<0.02) values were significantly lower in the RF-T group compared to the RF-C group. The ES was considered large for systolic and diastolic BP. Neither of the control groups showed any detectable changes after 16 weeks (p>0.02) (Table 2). 

**Table 2.** Hemodynamic variables at rest, heart rate variability indices, and high-sensitivity C-reactive protein levels of the groups studied.

**Variables**	**Trained**				**Control**				**Trained vs Control**	
	**Before**	**After**	**p**	**ES**	**Before**	**After**	**p**	**ES**	**p for before**	**p for after**
	**RF-T (n=10)**				**RF-C (n=10)**					
Height (cm)	169(7)	-	-		169(8)	-	-		-	-
Weight (kg)	79.2(8.8)	78.0(12.6)	0.03	0.26	74.0(21.0)	77.5(24.6)	0.30	0.05	0.73	0.73
BMI (kg/m2)	26.7(2.5)	26.1(2.7)	0.03	0.41	26.3(5.6)	27.5(5.4)	0.30	0.14	0.79	0.73
SBP (mmHg)	130(17.5)	120(7.5)	0.03	0.67	127(9.5)	130(11.2)	0.05	0.53	0.85	0.01
DBP (mmHg)	85(10)	80(3.7)	0.03	0.76	89(10)	88(8.7)	0.22	0.33	0.85	0.008
HR (bpm)	61(17)	67(11)	0.79	0.03	63(11)	66(14)	0.08	0.41	0.91	0.57
Spectral and symbolic indices										
LF (nu)	0.55(0.24)	0.46(0.24)	0.28	0.36	0.63(0.39)	0.73(0.07)	0.20	0.62	0.68	0.002
HF (nu)	0.44(0.24)	0.53(0.24)	0.28	0.36	0.36(0.39)	0.26(0.07)	0.20	0.62	0.68	0.002
0V%	29.2(20.2)	14.7(19.6)	0.009	0.67	25.0(10.5)	31.1(9.2)	0.24	0.22	0.63	0.01
2UV%	16.8(10.5)	24.2(14.3)	0.005	0.76	12.2(23.4)	10.3(6.1)	0.18	0.64	0.73	0.01
Inflammatory marker										
hs-CRP (mg/dL)	0.18(1.0)	0.04(0.05)	0.01	0.37	0.55(0.72)	1.15(1.02)	0.009	1.05	0.27	0.008
	CAD-T (n= 12)				CAD-C (n= 10)					
Height (cm)	167(12.2)	-	-		171(10.5)	-	-		-	-
Weight (kg)	82(23.7)	79(26.0)	0.03	0.28	86.5(16.4)	85.5(15.5)	0.93	0.02	0.82	0.65
BMI (kg/m2)	29.0(5.7)	29.3(4.9)	0.04	0.23	27.9(4.1)	28.4(6.4)	0.49	0.08	0.82	0.80
SBP (mmHg)	130(10)	120(2.5)	0.005	0.86	130(23.7)	140(17.5)	0.03	0.40	0.34	0.14
DBP (mmHg)	90(10)	80(2.5)	0.01	1.09	85(10)	90(10)	0.42	0.22	0.49	0.04
HR (bpm)	63(13)	72(12)	0.15	0.52	60(9)	57(6)	0.06	0.24	0.45	0.97
Spectral and symbolic indices										
LF (nu)	0.61(0.31)	0.58(0.13)	0.23	0.35	0.60(0.22)	0.55(0.21)	0.28	0.24	0.45	0.87
HF (nu)	0.38(0.31)	0.41(0.13)	0.23	0.35	0.39(0.22)	0.44(0.21)	0.28	0.24	0.45	0.87
0V%	35.7(29.6)	25.9(13.5)	0.09	0.50	22.5(14.2)	25.4(17.1)	0.07	0.61	0.10	0.82
2UV%	12.5(9.7)	19.0(13.0)	0.004	0.71	21.2(27.1)	16.2(13.1)	0.05	0.58	0.10	0.67
Inflammatory marker										
hs-CRP (mg/dL)	1.2(1.1)	0.7(0.6)	0.01	0.63	0.9(0.5)	1.1(0.4)	0.006	0.43	0.18	0.22

Data are presented as median (interquartile range). RF-T = risk factor-trained; CAD-T = coronary artery disease-trained; RF-C = risk factor-control; CAD-C = coronary artery disease-control; ES= effect size; BMI = body mass index; SBP = systolic blood pressure; DBP = diastolic blood pressure; HR = heart rate at rest; LF= low frequency; HF= high frequency; nu= normalized units; 0V% = percentage of pattern with no variation; 2UV% = percentage of pattern with two unlike variations; hs-CRP = high-sensitivity C-reactive protein. Signiﬁcance level of p<0.02.

At baseline, there were no significant differences in HRV indices (0V% and 2UV%; LFnu and HFnu) between trained and control groups. In the RF-T group, only 0V% (sympathetic modulation) values decreased after IT (p<0.02), while in the CAD-T group, LFnu and 0V% did not differ significantly at baseline (p>0.02). For both trained groups, 0V% presented medium ES. Moreover, only the RF-T group presented higher LFnu and 0UV% values than the RF-C group (p=0.01).

Regarding parasympathetic modulation, only 2UV% values increased significantly in the RF-T and CAD-T groups (p<0.02) after IT. For both trained group, 2UV% presented medium ES. Moreover, the RF-T group presented higher HFnu and 2UV% values than the RF-C group (p<0.02), while the HFnu and 2UV% values of the CAD-T and CAD-C groups did not differ significantly (p>0.02). None of the HRV indices in the control groups showed any significant changes after 16 weeks.

There were no significant differences among groups in hs-CRP level at baseline. After IT, the hs-CRP level in the RF-T and CAD-T groups diminished significantly (p<0.02), while in both control groups increased significantly (p<0.02). For the RF-T group, hs-CRP presented small ES, while in the CAD-T group, the ES was medium. Furthermore, the levels of hs-CRP (p=0.008) in the RF-T group were lower than those observed in the RF-C group.

### Analysis of covariance (ANCOVA) and linear regression

The linear regression examined the association of the use of medication and risk factors with the hs CRP levels and only symbolic indices. Use of beta-blockers accounted for 51% of the variance in 0V% in the RF-T group (p<0.05). In the CAD-T group, however, the use of beta-blockers and angiotensin-converting enzyme (ACE) inhibitors, dyslipidemia, and hypertension accounted for 65% of the variance in 0V% (p<0.05). Furthermore, the use of beta-blockers and ACE inhibitors as well as dyslipidemia accounted for 62% of the variance in 2UV% in the CAD-T group (p<0.05). The covariates that accounted for 50% of the variance in hs-CRP levels included PCI>3 months, use of ACE inhibitors, hypolipidemic agents, and dyslipidemia in the CAD-T group (p<0.05).

In the RF-C group, the use of beta-blockers, hypertension and obesity explained 69% of the variance in 2UV% (p<0.05). In the CAD-C group, dyslipidemia, hypertension, and obesity explained 64% of the variance in 0V% (p<0.05). All of the models satisfied the hypotheses of homoscedasticity and normality of residuals (Table 3). 

**Table 3.** Analysis of covariance (ANCOVA) and linear regression analysis of HRV indices and hs-CRP and the covariates for the trained and control groups.

**Groups**	**Dependent variables**	**Covariates**	**P**	**Intercept**	**Estimate**	**r^2^**
FT-T	0V%	Beta blocker	0.02	28.96	-14.69	0.51
CAD-T	0V%	Beta blocker	0.05	38.03	-17.43	0.65
		ACE inhibitor	0.03		-12.78	
		Dyslipidemia	0.005		10.14	
		Hypertension	0.01		18.41	
	2UV%	Beta blocker	0.02	9.78	10.74	0.62
		ACE inhibitor	0.05		11.22	
		Dyslipidemia	0.007		-5.65	
	hs-CRP	ACE inhibitor	0.04	1.17	-9.25	0.50
		Hypolipidemic	0.03		-0.18	
		PCI	0.01		-0.20	
RF-C	2UV%	Beta blocker	0.04	48.07	-3.41	0.69
		Hypertension	0.02		3.20	
		Obesity	0.05		3.15	
CAD-C	0V%	Dyslipidemia	0.02	48.24	20.41	0.64
		Hypertension	0.03		11.84	
		Obesity	0.04		0.85	

r^2^ = coefficient of determination; 0V% = percentage of pattern with no variation; 2UV% = percentage of pattern with two unlike variations; hs-CRP = high-sensitivity C-reactive protein; ACE = angiotensin-converting enzyme; RF-T = risk factor-trained; CAD-T = coronary artery disease-trained; RF-C = risk factor-control; CAD-C = coronary artery disease-control; PCI = percutaneous coronary intervention; ANCOVA and linear regression. Signiﬁcance level of p<0.05.

### Correlation between heart rate variability indices and C-reactive protein

A significant and positive correlation was found between 0V% (sympathetic modulation) and hs-CRP before (CAD-T, r=0.78; RF-T, r=0.85, with p<0.001) and after IT (CAD-T, r=0.80; RF-T, r=0.97, with p<0.001). With regard to 2UV% (parasympathetic modulation), a significantly relevant correlation was found with hs-CRP before (CAD-T, r=-0.74; RF-T, r=-0.78, with p<0.001) and after IT (CAD-T, r=-0.75; RF-T, r=-0.86, with p<0.001). Spectral indices did not correlate with hs-CRP (p>0.05).

## Discussion

This study demonstrated that IT based on VAT led to significant improvements in the autonomic modulation of HR as well as a reduction in hs-CRP levels. Furthermore, the present study showed that hs-CRP levels are positively associated with sympathetic modulation and negatively associated with parasympathetic modulation.

Notably, IT at the VAT level promoted an increase in parasympathetic modulation in both groups RF-T and CAD-T, while the reduction in sympathetic modulation was observed only for the RF-T group. Previous studies have shown that autonomic adaptions can be attributed to a reduction in efferent sympathetic neural outflow to the sinoatrial node^22^^,^ 23. Other mechanisms that result in an increase in vagal modulation through exercise training include the shear stress-induced up regulation of endothelial nitric oxide (NO) synthase. Recent research has demonstrated that nitric oxide, which is widely recognized as an endothelial control mechanism, is also a vagal modulator. In addition, NO exerts a facilitative effect on the afferent baroreflex and augments central and peripheral vagal neuronal activity^24^. Regarding methodological aspects of HRV analysis, although the symbolic analysis was more efficient in identifying changes in cardiac autonomic control than spectral analysis, this method combined with standard linear parameters can improve the performance of HRV analysis. Moreover, this finding was important for determining the applicability of symbolic analysis as a tool to evaluate autonomic adaptation-induced exercise training.

A significant decrease in CRP levels was observed in both trained groups. Particularly in the RF-T group, the decreases in the hs-CRP level indicated a reduced CAD risk after IT. The CAD-T group showed a greater reduction in the hs-CRP level than the RF-T group, which may suggest that high-risk individuals benefit more from IT than those with lower risk. In fact, this finding is consistent with previous observations showing that individuals with a high level of physical performance demonstrate lower hs-CRP levels than individuals with a sedentary lifestyle, regardless of gender and associated risk factors^25^.

Recent studies have shown that increased parasympathetic modulation may also play a role in mediating the inhibition of the inflammatory response observed in trained individuals^26^^-^ 28. The authors of these studies believe that one of the alternative mechanisms leading to reduced levels of circulating pro-inflammatory cytokines involves the cholinergic anti-inflammatory pathway^4^. In this pathway, acetylcholine inhibits the synthesis and release of pro-inflammatory cytokines by macrophages and other cytokine-producing cells by activating the alpha-7 nicotinic receptor (nAChRα7), which is expressed at the plasma membrane of these immune cells, resulting in reduced CRP levels^4^.

Singh et al.^29^ found a correlation between the linear indices of HRV, which reflect sympathetic modulation, and hs-CRP. These authors suggest that sympathetic activation promotes an increased concentration of circulating catecholamines, thereby stimulating the synthesis and release of pro-inflammatory cytokines. The adaptation of the sympathetic autonomic modulation of HR in the RF-T group, as shown by the reduced 0V% after IT, may also be considered an important pathway contributing to reduce CRP levels.

Analysis of the correlation between HRV indices and hs-CRP levels, adjusted according to clinical variables (angioplasty>3 months), the use of medications (beta-blockers, ACE inhibitor, and hypolipidemic agents), and cardiovascular risk factors (hypertension, dyslipidemia, and obesity), indicated that the decrease in hs-CRP levels after IT was negatively correlated with the 2UV% (which reflects the parasympathetic modulation of HR) and positively correlated with the 0V% (which reflects the sympathetic modulation of HR). These findings reinforce the notion of a cholinergic anti-inflammatory pathway, and the anti inflammatory properties of acetylcholine, and suggest that vagal modulation may inhibit the production of pro inflammatory mediators, and thus establishing the causality link between both measures.

With regard to BP, both trained groups presented a decrease in systolic and diastolic BP at rest after IT. Several mechanisms may be involved in the reduction in BP, such as decreased sympathetic tone in smooth muscle cells in the arterial wall and possible increase in NO bioavailability that decreases peripheral vascular resistance^24^^,^ 30.

It is noteworthy that the RF and CAD groups are different in relation to presence of risk factors and medication use. The same can be observed between the trained and control RF groups. Considering that cardiovascular risk factors and medication may affect the HRV and the hs-CRP, analysis of covariance and linear regression showed that cardiovascular risk factors, such as dyslipidemia, hypertension, and obesity, led to a significant negative effect on 0V% (sympathetic modulation) and on 2UV% (parasympathetic modulation) in the trained and control groups. Therefore, IT represents an important non-pharmacological therapeutic approach that should be included in the cardiac rehabilitation program of patients with CAD in view of its benefits in reducing risk factors and preventing the progression of CAD. On the other hand, medications such as beta-blockers and ACE inhibitors can attenuate vasoconstriction and sympathetic modulation, improving autonomic modulation of HR^31^. Hypolipidemic agents and ACE inhibitors have beneficial effects on hs-CRP because these drugs act on the control of lipid profile and hypertension, which are important risk factors to the development of atherosclerosis^32^.

The results of the present study may offer new possibilities in the field of cardiac rehabilitation, with respect to exercise training programs at VAT level. Cardiorespiratory parameters obtained at the VAT are of fundamental clinical importance to diagnosis, prescription of physical exercise, evaluation and re-evaluation of functional aerobic capacity, and monitoring of patients' development during exercise training programs. Furthermore, these results provide new insight into the main effects of IT on cardiac autonomic modulation and inflammation that could be expected in a larger sample. Thus, we can further develop the IT program based on VAT intensities as a new treatment protocol for cardiac patients.

### Study limitations

The autonomic adaptations promoted by the IT program are restricted to male patients with CAD and/or cardiovascular RF, which prevents us from generalizing these results to women and other cardiac diseases. Other limitations were the small sample and the fact that the present study did not include a group of healthy male subjects. The latter is especially important considering that the symbolic indices and hs-CRP present high inter-individual variability. Moreover, other inflammatory markers such as interleukin 6, fibrinogen, soluble intercellular adhesion molecule-1, and serum amyloid A, as well as echocardiogram were not measured. Furthermore, the CPET in cardiac rehabilitation centers still remains inaccessible, mainly due to the complexity and high costs of this technology. However, considering its importance, strong efforts are needed to encourage the use of CPET in exercise intensity assessment and prescription of cardiac rehabilitation programs.

## Conclusion

Interval training program based on VAT level led to improve in cardiac autonomic modulation, evidenced by increase in the parasympathetic modulation and decrease in the sympathetic modulation, associated with reduction of hs-CRP.
